# Building partnerships: A case study of physical activity researchers and practitioners collaborating to build evidence to inform the delivery of a workplace step count challenge

**DOI:** 10.3389/fspor.2022.1067127

**Published:** 2023-01-04

**Authors:** Ailsa Niven, James A. Ainge, Mary Allison, Trish Gorely, Paul Kelly, Gozde Ozakinci, Gemma C. Ryde, Simone A. Tomaz, Samuel Warne, Victoria Whiteford, Carl Greenwood

**Affiliations:** ^1^ Physical Activity for Health Research Centre, University of Edinburgh, Edinburgh, United Kingdom; ^2^ School of Psychology & Neuroscience, University of St Andrews, St Andrews, United Kingdom; ^3^ Department of Nursing and Midwifery, University of the Highlands and Islands, Inverness, United Kingdom; ^4^ Division of Psychology, University of Stirling, Stirling, United Kingdom; ^5^ BHF Glasgow Cardiovascular Research Centre, University of Kings College, Glasgow, United Kingdom; ^6^ Faculty of Health Sciences and Sport, University of Stirling, Stirling, United Kingdom; ^7^ Population and Behavioural Science Division, School of Medicine, University of St Andrews, St Andrews, United Kingdom; ^8^ School of Kinesiology, Faculty of Education, University of British Columbia, Vancouver, BC, Canada; ^9^ Paths for All, Stirling, United Kingdom

**Keywords:** worksite, evaluation, intervention, Scotland, collaboration

## Abstract

**Background:**

Walking is an integral part of Scotland's National Physical Activity Strategy, and the charity Paths for All's Workplace Step Count Challenge is a flagship programme within this strategy to promote physical activity. Effectively promoting physical activity requires collaborative engagement between stakeholders. However, there is limited guidance on how to do this. The aim of this case study is to share an example of a partnership between Paths for All and researchers to inform the development and delivery of the Workplace Step Count Challenge.

**Method:**

An overview of the partnership, example activities, reflections on opportunities and challenges, and suggestions for future partnership working are considered.

**Results:**

The partnership has evolved and strengthened over time through building trust. Many of the research activities provide an evidence base for the intervention. This work is mutually beneficial providing support for the work of the organisation, and opportunities for researchers to undertake “real world” research, leading to formal outputs and funding. The “real world” nature is challenging to integrate the most robust research designs. Recommendations for developing future partnerships were identified.

**Conclusion:**

Promoting physical activity effectively requires partnership working, and this paper provides insight into how such partnerships can work to inform future collaborations.

## Introduction

Physical activity has well-established physical and mental health benefits (1–3). However, large proportions of the population across the world are insufficiently physically active to reap these benefits (4, 5). Active transport (including walking, cycling and wheeling) and workplace settings have been identified as two of eight global investments that work to enhance physical activity behaviour (6). Indeed, evidence indicates that workplace interventions can be effective in increasing physical activity (7), and targeting walking is a promising strategy (8). Workplace “challenges” incorporating activity trackers to count steps are common initiatives to promote employee physical activity through increased walking, including active travel. One such challenge is Paths for All's Workplace Step Count Challenge (9), which is a flagship programme within Scotland's National Walking Strategy.

Scotland's National Walking Strategy (10) was launched with a vision to create “A Scotland where everyone benefits from walking as part of their everyday journeys, enjoys walking in the outdoors, and where places are well designed to encourage walking.” (p. 4). Consistent with global recommendations to support the optimal implementation of such policies (6), this strategy is operationalised in a wide-ranging action plan (11) working with partners across a range of sectors. The action plan highlights the importance of research to develop the evidence base for walking in Scotland, and to support the implementation of the strategy. The success of the action plan will depend on the development of effective cross-sector partnerships, such as collaborations between researchers and practitioners, who plan and deliver walking initiatives like the Workplace Step Count Challenge.

## Researcher-Practitioner partnerships

Partnerships between researchers and practitioners have been increasingly called for by funders, government agencies, policy makers (12), and physical activity advocates (6). Across different disciplines and geographical locations, these partnerships have been given different names (e.g., integrated knowledge translation, knowledge transfer and exchange, research-practice partnerships) (13). Nevertheless, they are all based on the assumption that collaboration between researchers and practitioners/policy makers/other research users will “enable and enhance both the use of research and increase the amount of research relevant to end users” (12, p. 2). Indeed, these partnerships have been shown to have mutual benefits for both researchers and practitioners. For example, the collaborations may bring together different perspectives and expectations, which can lead to a broader understanding of needs and contextual influences, the identification and use of appropriate research methods for a context, and support routes to dissemination, and change of practice. Further, it has been suggested that the users of research benefit through increased awareness of relevant research, reflection on their own activities from different perspectives, and enhanced skills (13). Additionally, researchers benefit through a more nuanced understanding of the real world environment, the development of research questions with real world applicability, and through conversations about the interpretation and meaning of findings as they relate to real world situations (13). Given these mutual benefits, the research itself has greater potential for impact.

Nystom et al. (2018) (12) identified three main strategies to build or enhance research partnerships which are distinguished by who is driving the relationship, and these may change over time. In push strategies the relationship is driven by the researchers, which contrasts with pull strategies that are driven by the needs and demands of research users (14). The third strategy, linkage and exchange, is co-production of applied research useful for both parties (15).

Within physical activity research, this third strategy is reflected in Estabrooks and colleagues (16) proposal for collaborative working between researchers, practice professionals and decision makers to maximise the public health potential of physical activity interventions. Similar to others, they argue that the development of a mutual understanding of the value of different types of evidence and an acknowledgement of the unique knowledge, skills, and experiences that different collaborators bring to a project, mean that practitioners can act more readily on the best available evidence. Estabrooks et al. go on to describe an Integrated Research-Practice Partnership Practice Model (IRPPPM), which is based on an “iterative process used to co-produce research-based and practice-relevant evidence” (16, p. 4). Fundamental to this process is an emphasis on collaboration between practitioners, decision makers and researchers, and practicality with a move away from “push” strategies for evidence-based interventions, which may meet with resistance.

Research exploring research-practice partnerships has typically focused on the outcomes, and there has been less focus on the activities that characterise the partnerships. For example, there have been calls for researchers to capture and report on the nature of partnership activities; who is involved in what?; and how does it function? (13, 17). There are some examples reflecting on these collaborations in health care, educational, and community participatory research, but there are limited examples within the physical activity domain (e.g., 16, 18). Therefore, the overall aim of this paper is to illustrate as an example the development and strengthening of a partnership between the organisation Paths for All and the local research community to work collaboratively in building the best available evidence to inform the development and delivery of the workplace Step Count Challenge. The specific objectives are to share examples of partnership research activities, highlight the opportunities and challenges of undertaking research in a “real world” setting, and reflect on what we have learned that may be transferable to other settings and partnerships.

## Setting the context: Paths for All and Workplace Step Count Challenge

Paths for All (PFA) is a Scottish charity whose vision is for a happier, healthier Scotland where physical activity improves quality of life and wellbeing for all. PFA's aim is to significantly increase the number of people who choose to walk for leisure or travel, to create better environments for walking, wheeling and cycling, and to influence policy at all levels [e.g., through the development of/contribution to policy documents including The National Walking Strategy (10)] to have an increased focus on physical activity. PFA receives Scottish Government funding to carry out this work.

PFA's Step Count Challenge (SCC) (9) is a flagship programme of the National Walking Strategy for promoting walking as an important part of the working day. The SCC was launched in 2011 to support workplaces to encourage staff to move more in-and-around the working day, and was designed to complement Public Health Scotland's Healthy Working Lives Award (19). The SCC is an online team-based walking challenge that has evolved over the last ten years to enhance the participant experience, and functionality of the interface. PFA has worked with users at each stage to make improvements based on feedback and delivered pilot challenges with stakeholders to test and review changes before launching.

In the 2021 delivery, participants registered on the SCC website in teams of five and paid £30 per team to participate. During the challenge, participants recorded their activity through a personal online dashboard; this activity can include walking, cycling, wheeling, running, swimming and yoga, and participants can also manually convert additional activities to steps. Activity data is added manually from participants own activity monitor, or by synchronizing with a selection of apps (Strava, Google Fit and Fitbit). Based on their recorded activity, participants are set tailored step-goals that increase as the challenge progresses. Participants can track and monitor their activity data and view leader boards that show how their total team step-count compares to others nationally. PFA provides update emails, competitions, prize draws, and blog posts on a range of topics.

The challenge runs twice a year, with an eight-week spring challenge and shorter four-week autumn challenge. It is open to workplaces from all sectors and they can register any number of teams. Workplaces can also set up bespoke challenges for their workplace at any time, with the workplace taking on the role of providing updates to participants during the challenge. In recent years the key messages of the SCC have focused on supporting participants to be active during the working day (e.g., through active meetings, taking regular desk breaks, etc.), promoting the mental health benefits of walking and being outdoors, and connecting with teammates either in person, or virtually.

Since 2011, PFA has delivered 19 national challenges and 78 bespoke challenges (introduced in 2016). The spring challenge generally attracts around 4,000 participants and the autumn challenge 2,000 participants. In financial year 2020/21 there were over 10,000 participations in SCC. With Covid-19 and the move to homeworking, there has been an increase in demand for bespoke challenges over the winter and spring of 2020/21 as workplaces look for ways to support staff whilst they are working remotely, and the challenge is seen as a tool to accomplish this.

## Researcher-practitioner partnership on the Workplace Step Count Challenge

The partnership includes PFA staff and researchers based at five Scottish higher education institutions, including the Universities of Edinburgh, Glasgow, the Highlands and Islands, St Andrews, and Stirling, often working collaboratively. The initial partnership was established more than ten years ago, and has evolved to include additional institutions. Collectively this partnership has included a number of research projects focused on the SCC. Table 1 provides an overview and synthesis of example research studies undertaken or ongoing as part of the partnership. The table illustrates the main research question, methods adopted, summary findings, study type, and outputs. These research studies have been undertaken both in response to requests from PFA and proposals from the universities to PFA, and were all approved by respective institutional ethical committees.

**Table 1:** Example research studies undertaken by the partnership.

Study focus	Method	Summary findings	Study type	Outputs
1. The impact of COVID-adapted SCC on mental well-being	Mixed-methods (quantitative questionnaires pre and post and qualitative interviews)	Enhanced well-being through: being outside and connecting with nature; it provided a distraction; mindfulness; social interaction	MSc student project	PFA blog—(20)
2. Evaluate the effect of 8-week SCC on physical activity behaviour and motivation using validated measures	Pre-post questionnaire design (within participant design)	Small changes in weekly walking, including walking for transport and leisure, but not at work. There were no significant changes in the other PA domains, with the exception of a reported decrease in sitting behaviour. Participants became more confident in walking, and were more autonomously motivated	Researcher project (internal funding)	Report to PFA (21), Infographic (see Figure 1), and video with PFA to communicate the findings to a wider audience (22). Peer review publication on motivation data (23).
3. Realist evaluation of SCC: How does a workplace walking programme produce its effects	Realist methodology: programme theory building using interviews and realist review, case studies to refine.	The process of “step counting in a workplace group” is a balance between personal goals and group dynamics. High levels of physical activity are generated from having fun, participating in a competition, and challenging oneself to do more. In other contexts, goal focus and group pressure can generate stress and/or drop out.	PhD student project (external funding ESRC SGSSS)	PhD Thesis (24) PFA podcast (25) and blogs (e.g., 26)
4. Evaluate the effect of 8-week SCC on changes in step-count across four years of delivery using routinely collected data	Quantitative analysis of routinely collected SCC data	Across the four years there was a largely consistent increase in step counts at each week compared with week 1. By week 8, participants had increased their steps by on average 906 steps per day	Researcher-led project (across 3 institutions) (unfunded)	Peer-reviewed publication (27). PFA news item (28)
5. What are the psychological determinants and consequences of participation in SCC?	Qualitative interviews	Main motives: incorporating more physical activity into their lives, and improving their fitness. Perceived benefits: weight loss, enhanced muscle tone, feelings of vigour, dedicated time to enjoy nature either by themselves or with others, the opportunity to be with own thoughts.	MSc project	Forthcoming
6. Multidisciplinary approach to quantifying the physical and mental health benefits of participating in SCC	Quantitative online survey at multiple time points Experimental studies	Ongoing analyses are examining links between physical and mental health with SCC participation. Forthcoming research to consider the link between SCC participation and cognitive function	PhD student project (external funding ESRC SGSSS)	Forthcoming
7. Evaluating the beneficial effects of the 4-week SCC on work-related outcomes, and highlighting challenges of “real world” research	Quantitative pre, week 1 and post SCC questionnaire	Preliminary data suggest positive changes in step, stress and productivity	Initially UGT project, then further developed (unfunded)	(29)

It is notable that a common theme across the studies has been to evaluate the effectiveness of the SCC on different outcomes. Studies have focused on documenting changes in physical activity (including steps) and also other health (i.e., cognitive, mental, physical), and work-related outcomes. More recent research has focused on developing a more nuanced understanding regarding for whom the SCC is effective (e.g., studies 3 & 6). Research activities are undertaken by established researchers, and also by undergraduate, postgraduate and doctoral level researchers, employing both quantitative and qualitative methods. A range of outputs have been produced, including both academic (e.g., peer-reviewed articles, conference presentations) and more external facing materials (e.g., blog posts, infographics), all detailed in Table 1. This collective research effort is building the evidence for the beneficial effects of the SCC.

## The opportunities from the researcher-practitioner partnership

From the perspective of PFA there have been a number of opportunities and benefits from the evolving research-practice partnership. As part of ongoing monitoring and evaluation for reporting to funders and to support ongoing improvements and efficiencies to the SCC experience, PFA collects a range of data and feedback from participants. Working with the universities in this partnership has created human capacity to explore in greater depth the experiences and outcomes of SCC participants. These activities have contributed to a deeper understanding of how the SCC works and for whom, including insight into participant's motivation and barriers, and team dynamics. These insights have directly led to improvements around the SCC platform, communications, messaging and design. Researchers have also provided informal assistance to test platform changes. Furthermore, researchers have contributed to blogs providing a different voice for SCC participants to encounter. Other less obvious benefits that PFA have noted include (1) the advocacy and awareness raising that occurs through academic presentations, posters, publications and networking; and, (2) the research partnership helping to demonstrate the impact of PFA's work thus supporting the case for future funding and resource. Finally, PFA view the partnership as a gateway to opportunities for further research collaborations to support their broader work. For example, researchers within this partnership have brought together other research colleagues to support a proposal for work with young people.

**Figure 1:**
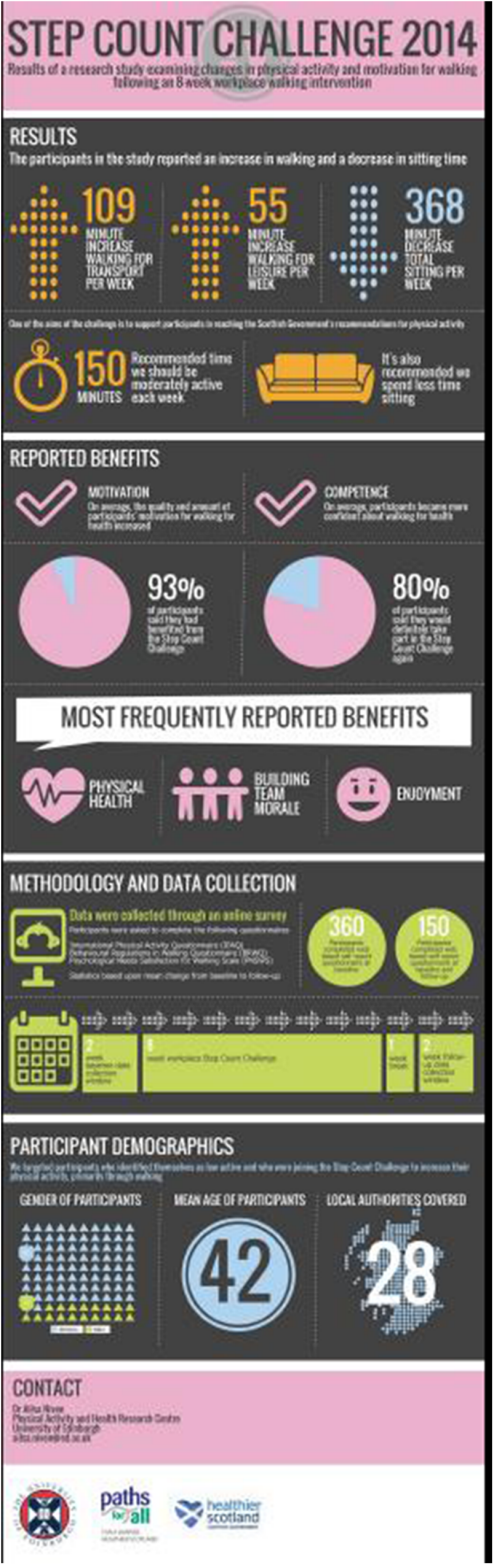
Example infographic reporting of research findings.

For researchers the opportunities from this collaboration are multiple. As illustrated in our examples, there is a mutually beneficial opportunity from this partnership to facilitate UGT, PGT and PhD student projects that can address and build evidence for areas of practice identified by PFA. Students benefit greatly from working on a “real world” project, and although for UGT and PGT the scale of work is unlikely to lead to a peer reviewed publication, in addition to completing their studies, students are typically supported to create an output that is accessible to a more lay audience. The experience of working with practitioners early on in their training also provides valuable opportunities for networking and skill development for establishing professional relationships. Importantly, outputs produced as part of these projects provide PFA with useful materials to share with funders and participants. Larger scale PhD and staff projects have the potential to lead to peer-reviewed publications, and the opportunity to collect data with PFA is of considerable benefit to researchers in undertaking this key aspect of their jobs. In addition to peer-reviewed publications, working with PFA and their communications experts has resulted in scientific communication outputs that are more effective in disseminating the findings more widely (see Table 1, and Figure 1 for example). Such outputs are important in facilitating the impact of the research findings, which is increasingly recognised as a key indicator of research effectiveness (30).

As is evident throughout our descriptions of research, although some research is unfunded we have also been successful in securing both internal and external funding to support our collaborations. Having an enduring relationship with Paths for All, and their support in engaging with our research activities is certainly advantageous in applying for research funding. This funding enables us to dedicate further time to support the activities of the partnership, and ultimately produce outputs that are optimally useful to PFA. In such projects, more formal collaborative agreements between the institutions and PFA are implemented to address financial and legal requirements.

A final opportunity that as a collective we wanted to document, is that working together is rewarding. We do work truly pragmatically and collaboratively to find solutions to make the research happen. For example, during COVID-19 PFA supported an extension to their online portal to accommodate PhD data collection that facilitated the process in a seamless way. As researchers, we also benefit from working cross-institutionally, sharing discipline and methodological expertise around a common interest. This collaboration facilitates sharing of learning, avoidance of duplication, identification of gaps in knowledge, and working collectively to address challenges.

## The challenges of the researcher-practitioner partnership

For PFA the main challenges of the partnership relate to ensuring that the participants are informed about why the research is being undertaken, and that the research activities are easy to engage in without additional burden.

The main challenges identified by researchers reflect more the challenges in undertaking research in the “real world”, rather than necessarily the operationalising of the partnership. As Ryde et al. (29) also outline, challenges of collecting workplace outcomes during the SCC included low initial recruitment rates, poor compliance to data collection and lack of true baseline. For example, across our studies recruitment of participants has been challenging with a relatively small proportion of SCC participants choosing to take part in the research. In studies 2 and 7 listed in Table 1, recruitment rates were 10% and 12%, respectively. These challenges may in part be due to the inclusion of robust research measures, which can add participant burden. Participant attrition is a further challenge, when aiming to collect data over time. This leads to the problem of incomplete data sets due to instances of missing data points. Researchers then have to decide whether to use statistical methods to impute data from the sample or analyse only those participants who provided data for all time points. Although these recruitment and attrition challenges do occur and are common issues in real-world data collection, it is notable that working with PFA has been important to achieve even these levels of participation. For example, PFA support recruitment through endorsement, integrating data collection into existing systems, communications, and incentivising participation through the provision of “spot” prizes.

A further challenge that has limited the conclusions that can be drawn from studies, has been the absence of a true “baseline” in the online recording of steps by participants engaging in the SCC (27, 29). Typically, the first reported data has been from week 1, which may be elevated by initial enthusiasm, and mask the true effect of the SCC. Recommendations have been made to Paths for All about integrating research more effectively into real-world interventions, such as including a true baseline as part of the intervention itself and not just for research purposes, enhancing routinely collected data to include additional outcomes, and to automatically transfer this routine data (with relevant permissions) to reduce participant burden (29). However, this should not be to the detriment of the delivery of the intervention itself, where the primary objective is to increase physical activity.

An additional challenge, relates to the reliability and validity of the assessment of step counts. In all of the studies listed in Table 1, the researchers have relied on participants reporting their steps as assessed by their own device. Although commonly used “fitness trackers” are becoming increasingly sophisticated and robust (31), the research would be strengthened by being able to standardize measurement across participants. However, the resource and time required to do that is not always available, and can impact participant recruitment.

Ultimately, scaffolding the most robust research design around an ongoing programme is very difficult. For example, due to ethical, logistical and financial issues it is rarely possible to recruit a control arm to the study where characteristics of the individuals could be matched (e.g., age, gender, physical activity levels) to enhance the internal validity of the study. Therefore, from a perspective informed by a positivist bio-medical model, the research quality is compromised. However, as demonstrated by Allison (24), realist methodology (32, 33) offered the possibility to develop, refine and test a programme theory for the Step Count Challenge. Such theory has provided insights into how the SCC works, for whom, in what context, and why. Having this refined programme theory has helped clarify how and why this programme works and offers a new opportunity for others to test all or some of these theories, using a positivist bio-medical model.

## Reflections, next steps, recommendations, and conclusion

Paths for All has worked with a range of researchers and academics for 10 years focusing on the SCC. The early phases of this collaboration were very much driven by individual researchers approaching PFA with ideas for research (“push” strategies); however, over time the relationship has become much more one of linkage and exchange, with questions and needs being identified through discussions with PFA and linking individual research teams together. This natural evolution reflects the different stages of research partnerships identified in the literature (12). This shift was at least partially driven by the perceived utility of the early work to help PFA gain a deeper understanding of the benefits of the SCC works.

We hope that this paper highlights the benefits of researchers and practitioners working together in partnership to undertake research that will address pertinent issues, and impact on practice. In preparing this paper, we have individually reflected on “why we do it” (i.e., work collaboratively). Whilst there are extrinsic benefits in terms of producing outputs, funding and evidence of impact, a key theme evident in our reflections, related to more intrinsic drivers. Specifically, the collaboration is fun and enjoyable, where as a collective we have a level of mutual respect for all that each partner brings to the collaboration, including students, researchers and PFA staff. This level of respect and trust has taken time to develop. We work hard to ensure open and regular communication during projects, and identify and articulate clear expectations that explicitly accommodate the needs of all parties. We then work hard to deliver on those outcomes, and as researchers ensure we “close the loop” and provide our partner with useful outputs.

As we move forward, we now plan to build on and formalise the collaborative partnership to work together to generate, evaluate and translate relevant evidence. This group will seek to enhance synergy and coherence in the way evidence informs the SCC. At the time of writing we are developing our Statement of Purpose (e.g., who, why, when, what), and have plans to enhance the visibility of the partnership through a web-presence to highlight our work, and to make sure the partnership is inclusive of other interested researchers. Such a forum will also support PFA's desire to effectively coordinate the different projects and ensure there is equity and transparency in the allocation of resources and opportunities.

Based on our reflections on the partnership and the opportunities and challenges we have encountered, we have identified some key recommendations that may help others who are developing new partnerships. Firstly, try not to force the partnership, and be mindful that it will take time (i.e., years not months) to establish trusting relationships. Secondly, aim to have clear roles and expectations within the partnership. Again, it will likely take time for expectations between partners to align, and be realistic as two different sectors come together. Thirdly, be prepared to compromise. For example, for researchers, it will rarely be possible to implement a highly controlled or randomised research design. For practitioners, research is rarely a quick process with rigorous processes required prior to, during, and after data collection. Finally, be mindful that the partnership should be mutually beneficial, and work together to ensure that each partner's needs are met. For example, for practitioners ensure that time is built into project planning to allow incorporation of research, and for researchers make sure to “close the loop” and deliver on promised feedback to practitioners and stakeholders.

To conclude, it has been recognised that collaborative partnerships are needed to effectively promote physical activity (6), and this paper has contributed to the literature by providing a specific example of how a research-practitioner partnership can work in the area of workplace physical activity. The paper has addressed a recognised gap in this area by focusing on who is involved in the partnership, and how it functions, rather than a sole focus on the outcomes of the research (13, 17). We hope that these insights, reflections and recommendations can support other researchers and practitioners in building fruitful collaborations that can enhance the relevance and impact of research activities.

## Data Availability

The original contributions presented in the study are included in the article/Supplementary Material, further inquiries can be directed to the corresponding author/s.
